# Influenza H5/H7 Virus Vaccination in Poultry and Reduction of Zoonotic Infections, Guangdong Province, China, 2017–18

**DOI:** 10.3201/eid2501.181259

**Published:** 2019-01

**Authors:** Jie Wu, Changwen Ke, Eric H.Y. Lau, Yingchao Song, Kit Ling Cheng, Lirong Zou, Min Kang, Tie Song, Malik Peiris, Hui-Ling Yen

**Affiliations:** Guangdong Provincial Center for Disease Control and Prevention, Guangdong, China (J. Wu, C. Ke, Y. Song, L. Zou, M. Kang, T. Song);; University of Hong Kong, Hong Kong, China (E.H.Y. Lau, K.L. Cheng, M. Peiris, H.-L. Yen)

**Keywords:** avian influenza, zoonoses, H7N9, H5/H7 poultry vaccine, One Health, influenza, viruses, Guangdong, China, vaccines, vaccination, respiratory infections

## Abstract

We compared the detection frequency of avian influenza H7 subtypes at live poultry markets in Guangdong Province, China, before and after the introduction of a bivalent H5/H7 vaccine in poultry. The vaccine was associated with a 92% reduction in H7 positivity rates among poultry and a 98% reduction in human H7N9 cases.

Human infections with avian influenza A(H7N9) virus have been documented in China since 2013 (*1*). Among 1,220 confirmed H7N9 case-patients during 2013–2017, a total of 73% reported poultry exposure, and 57% had visited live poultry markets (LPMs) before symptom onset (*2*). Because of the lack of apparent clinical signs in poultry infected with low pathogenicity H7N9 influenza virus, it has been challenging to rapidly identify and remove infected poultry at the LPMs or farms and to justify implementation of mandatory vaccination of poultry against this virus. Interventions such as market closure during human epidemics have temporarily reduced human exposure to live poultry and decreased zoonotic infection risk (*3*). However, the effect was not sustainable because H7N9 viruses continue to circulate within the LPM supply chain, leading to recurrent waves of human infections in winter months (*4*). 

During winter 2016–17, the H7N9 virus evolved into highly pathogenic avian influenza (HPAI) virus by acquiring a polybasic amino acid motif at the hemagglutinin cleavage site, rendering the virus capable of disseminating systematically and causing high mortality rates among chickens (*5**,**6*). In response to the emergence of HPAI H7N9 virus, the government of China amended the mandatory vaccination regimen for avian influenza in summer 2017. Specifically, a newly developed bivalent H5 (Re-8, based on clade 2.3.4.4 H5N1 virus A/chicken/Guizhou/4/2013) and H7 (Re-1, based on H7N9 virus A/pigeon/Shanghai/S1069/2013) vaccine replaced the previous bivalent H5 vaccine that targeted H5 clades 2.3.4.4 (Re-8) and 2.3.2.1 (Re-6, based on H5N1 virus A/duck/Guangdong/S1322/2010). The new bivalent H5/H7 vaccine was first introduced in Guangdong and Guangxi Provinces in July 2017; other provinces adopted the poultry vaccine by winter 2017–18. The vaccine coverage rate reported in November 2017 in Guangdong was 97.9% (282 million birds) among the target poultry population (*7*), which encompassed chickens, ducks, geese, quail, pigeons, and rare birds in captivity (*8*); however, the reported vaccine coverage varied in different provinces (*8*). Layers and breeders received 2 doses of the H5/H7 vaccine, whereas broilers sold within 70 days received 1 dose (*8*). 

To assess the effect of poultry vaccination on H7N9 prevalence in poultry and the subsequent effect on human zoonotic infection risk, we analyzed the temporal distribution of monthly H7N9 detection rates at LPMs and of human H7N9 cases in Guangdong Province during 2013–2018. We estimated the effect of the bivalent H5/H7 vaccine using a Poisson regression model.

## The Study

During January 2013–June 2018, a total of 22 collaborating laboratories collected 81,984 environmental samples from 345 retail LPMs and 24 wholesale LPMs distributed in 21 cities in Guangdong Province (*9*). Samples included poultry fecal droppings, drinking water, and various surface swab specimens from cages, chopping boards, display tables, and defeathering machines. Samples were stored in virus transport media (Zijian Biotech, Shenzhen, Lang Shan, China). Influenza A virus RNA segment 7 (M gene) was detected by quantitative real-time reverse transcription PCR (RT-PCR); positive samples underwent further testing to detect H5, H7, or H9 viral RNA using subtype-specific primers and probes (*10*). Human H7N9 infections were reported from 28 hospitals and 22 collaborating laboratories in Guangdong Province and verified by the Guangdong Provincial Center for Disease Control and Prevention by RT-PCR, virus isolation, or both (*10*).

**Figure 1:**
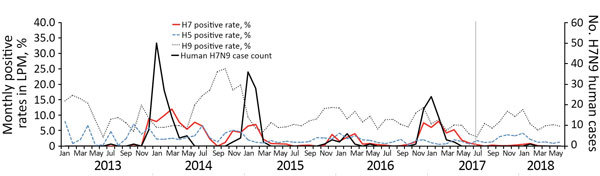
Monthly H5, H7, and H9 positive rates at live poultry markets (LPM) and human H7N9 cases in Guangdong Province, China, January 2013–June 2018. Vertical gray line shows the introduction (July 2017) of the bivalent H5/H7 vaccine in poultry.

During the 66-month period of the study, influenza A viral RNA was detected frequently from LPMs; the median monthly positive rate was 22.0% (range 7.4%–48.3%). H9 was the most frequently detected subtype; the median monthly positive rate was 12.1% (range 4.4%–37.6%) (Figure 1). H5 subtype was detected every month at low frequency (median monthly positive rate 3.1% [range 0.3%–11.9%]), whereas H7 subtype was detected at variable frequency (median monthly positive rate 0.6% [range 0%–12.0%]).

The bivalent H5/H7 vaccine was introduced in Guangdong in July 2017. We expected to see its effect on H7 prevalence in poultry and the accompanying human infections after September 2017 because of the 45-day fattening period for broilers before they are traded to the LPMs (*11*). The median H7 positive rate detected at LPMs before (January 2013–August 2017) the introduction of the bivalent H5/H7 vaccine was 0.8% (range 0%–12%). The rate after introduction (September 2017–June 2018) was 0.1% (range 0%–0.8%; p<0.001 by *t*-test). The median H5 positive rates detected during the same periods were 3.1% (range 0.3%–11.9%) before introduction and 2.7% (range 1.4%–6.3%) after (p = 0.727 by *t*-test). These results suggest that replacing the bivalent H5 vaccine that targeted H5 clades 2.3.4.4 and 2.3.2.1 with the bivalent H5/H7 vaccine that targeted H5 clade 2.3.4.4 alone did not significantly affect the H5 positive rate at LPMs during winter 2017–18.

**Figure 2:**
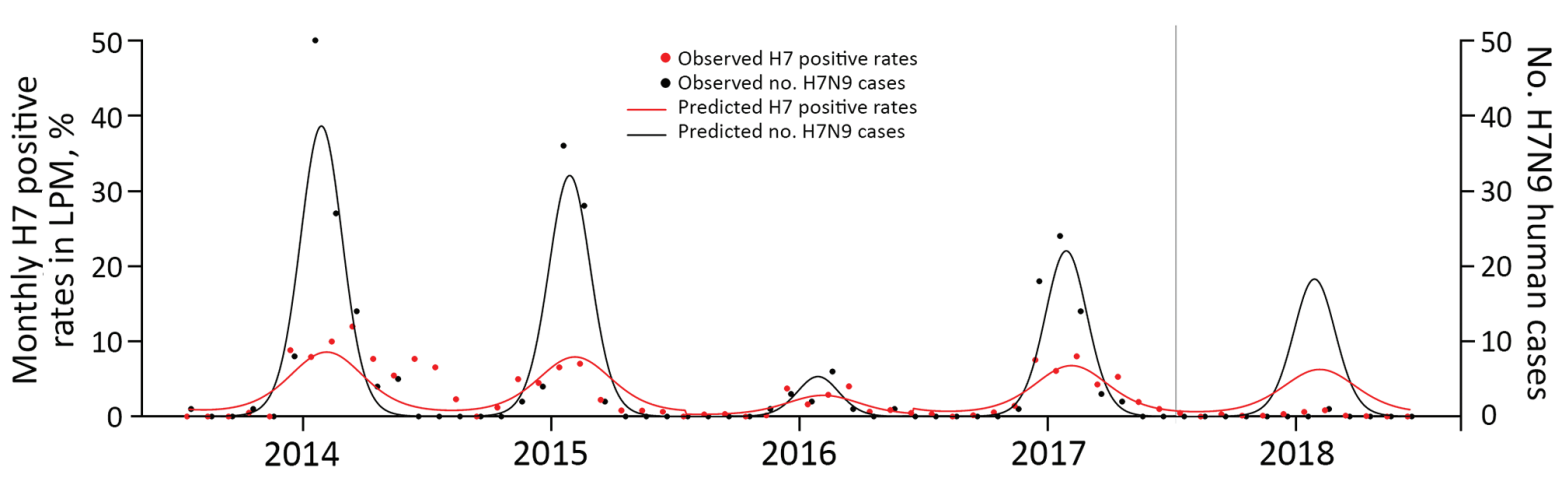
Observed and predicted monthly H7 positive rates at live poultry markets (LPM) and number of H7N9 human cases in Guangdong Province, China, July 2013–June 2018. Vertical gray line shows the introduction (July 2017) of the bivalent H5/H7 vaccine in poultry.

To further evaluate the effect of the bivalent H5/H7 poultry vaccine, we estimated the expected H7 positive rate at LPMs and the number of human H7N9 cases in Guangdong Province after September 2017 if the vaccination had not been implemented. Using baseline data collected before the vaccination period (January 2013–August 2017), we fitted separate Poisson regression models that described the risk for H7 positive detection in LPMs and of identifying human H7N9 cases in Guangdong Province, allowing for a long-term time trend, annual seasonal patterns, and lower H7N9 virus activity during winter 2015–16. This baseline model accounts for the potential effects of other intervention measures, including market rest days, which were similarly implemented in 2017–18. We plotted the predicted values during the vaccination period (Figure 2) and fitted another model for the full period (July 2013–June 2018) to test the vaccination effect by including an indicator variable for the postvaccination period. We estimated relative risks (RRs) and corresponding 95% CIs by fitting the model to data. The bivalent H5/H7 vaccine was associated with a reduction of 92% (RR 0.079, 95% CI 0.057–0.106) in the H7 positive rates at LPMs and 98% (RR 0.021, 95% CI 0.001–0.096) in the number of human H7N9 cases.

## Conclusions

Emergence of H7N9 highly pathogenic avian influenza virus during the fifth epidemic wave during winter 2016–17 has prompted the mandatory use of a bivalent H5/ H7 inactivated influenza vaccine in domestic poultry in China. Before then, a bivalent H5 poultry vaccine was used. Our results provided quantitative confirmation for the significant impact of the vaccine in reducing H7 detection frequency at LPMs and the corresponding reduction in human H7N9 infections in Guangdong Province, 1 year after implementation of the vaccine. The focus should be on achieving a high vaccine coverage rate in domestic poultry in which the H7N9 virus has been detected, recognizing that antigenic drift variants that escape vaccine-induced immunity may emerge. A second concern is whether H7N9, which predominantly infected chickens, may adapt to aquatic poultry, such as ducks. Such an event would prove a major challenge for the control strategy.

In conclusion, our analyses of longitudinal surveillance data support the association between the introduction of the bivalent H5/H7 vaccine for poultry and the reduction in zoonotic H7N9 disease. These results illustrate an example of combating zoonotic avian influenza virus at its source in a One Health approach.
